# Prediction of China’s Population Mortality under Limited Data

**DOI:** 10.3390/ijerph191912371

**Published:** 2022-09-28

**Authors:** Zhenmin Cheng, Wanwan Si, Zhiwei Xu, Kaibiao Xiang

**Affiliations:** 1 School of Mathematics and Statistics, Guizhou University, Guiyang 550025 , China; 2 School of Management, Guizhou University, Guiyang 550025 , China

**Keywords:** population mortality, stochastic mortality modeling, Lee-Carter, longevity risk

## Abstract

Population mortality is an important step in quantifying the risk of longevity. China lacks data on population mortality, especially the elderly population. Therefore, this paper first uses spline fitting to supplement the missing data and then uses dynamic models to predict the species mortality of the Chinese population, including age extrapolation and trend extrapolation. Firstly, for age extrapolation, kannisto is used to expand the data of the high-age population. Secondly, the Lee-Carter single-factor model is used to predict gender and age mortality. This paper fills and smoothes the deficiencies of the original data to make up for the deficiencies of our population mortality data and improve the prediction accuracy of population mortality and life expectancy, while analyzing the impact of mortality improvement and providing a theoretical basis for policies to deal with the risk of longevity.

## 1. Introduction

At present, China’s population aging has entered a stage of rapid development. At the same time, with the rapid advancement of aging, actively coping with population aging has become a national strategy [1]. In 2021, the results of the seventh national census showed that in 2020, the number of people over 65 years old in China reached 190.64 million, accounting for 13.5% of the total population, belonging to the “aging society”. The World Health Organization defines societies with 7%, 14%, and 20% of the population over 65 as “aging society”, “aging society” and “super-aging society”, respectively. According to the research data released by scholars from the global change data Lab (GCDL) in the UK, more than 20% of China’s population over the age of 65 will enter the “super-aging society” around 2035 [2].

In China’s current economic situation, the demographic dividend should be promoted to a quality-based upgrade, human capital potential should be tapped [3]. To further develop the demographic dividend, we must focus on population aging and on research on newborn life expectancy. In essence, the causes of China’s population aging include two aspects: the reduction of birth rate and the extension of average life expectancy. The reduction of birth rate leads to the decline of the proportion of the young and middle-aged population, the extension of average life expectancy, and the decline of mortality, resulting in the rapid growth of the aging population, and finally leading to the aging problem of China’s population [4]. Life expectancy is an important factor affecting population aging. According to the information released by the China Health Commission, from reform and opening up to 2019, the average life expectancy of China’s population increased from 67.9 years to 77.3 years. With the continuous extension of life expectancy, both economic subjects, life insurance companies, and individual families are suffering from the impact of longevity risk to varying degrees. Mortality prediction is not only the basis of life expectancy prediction but also an important standard to measure the risk of longevity.

Population mortality projections usually include two methods: one is to calculate population life expectancy by projecting sub-age mortality, the other is to calculate sub-age mortality from life tables by projecting population life expectancy [5].The mortality prediction model is mainly developed in the early static model (Makeham model, Gompertz model, H-P model) that describes the change of mortality with age to the classical dynamic model including time and age, such as the Lee-Carter model [6] and CBD [7] model. These are the most basic and classical of mortality prediction models. Later, with the progress of the economy and technology and the constant change of the evolution law of mortality, researchers continuously studied the prediction model of mortality according to the characteristics of actual data. Improvements are mainly made in two aspects: parameter estimation as well as model form. In terms of parameter estimation, Wilmoth [8] proposed weighted minimum and multiplication to solve the parameters of the Lee-Carter model; Lee and Miller [9] put forward three methods to improve the prediction of the Lee-Carter model: selecting fitting period, adjusting period factor with life expectancy, and eliminating “jump error”; Brouhns [10] used Poisson regression to estimate the parameters; Li [11] proposed Lee-Carter model and parameter estimation method under limited data. In terms of the model form, Booth [12] pointed out that the SVD method used in the Lee-Carter model can only extract the first term of a large number of age or time factors, and can expand the model to a complex form with multiple items, and use Poisson regression to estimate parameters. Renshaw and Haberman [13] used the extended form of the two-item Lee-Carter model to predict the mortality rate of British men. Spreeuw presents a mortality model where future stochastic changes in population-wide mortality are driven by a finite-state hierarchical Markov chain, it permits an exact computation of mortality indices [14]. Poisson regression and ARIMA were used to estimate and predict the parameters. Baran [15] used the trinomial Lee-Carter model to predict the mortality rate in Hungary.

Under the background of limited data in China, domestic scholars have also conducted a lot of research on mortality prediction models. There are two main adjustments to mortality prediction models. One is to adjust the parameter estimation methods. The common methods are singular value decomposition, least-square estimation, maximum likelihood estimation, and so on [16,17,18]. Li [19] extended the Lee-Carter model with the MCMC method under limited data conditions and estimated and predicted mortality in the Chinese Mainland with the help of mortality information in Taiwan and Japan. Wang and Zhao [20] defined the improvement factor of mortality under limited data and analyzed the random fluctuation of mortality of the elderly population by the Montecarlo method. Hu [21] used the MCMC method to estimate the parameters of the Lee-Carter model and believed that the Bayesian method effectively reduced the impact of data quality. Wu, Luo, Su, and Gao [22] readjusted the estimated parameters of the mortality model and further analyzed the longevity risk. The second is to focus on the formal expansion of the model, such as the number of expansion factors, the effect of joining the queue, and the expansion of the multi-population model. Hu [23] investigated and compared the prediction effect of the two-factor Lee Carter model The test results show that the goodness of fit and dispersion information criterion DIC of the two-factor model is significantly better than the single-factor model, and better grasps the volatility of mortality over time. Huang and Wang [24] used the Lee-Carter model with cohort effect to fit and predict male mortality. Zhao and Wang [25] used the multi-population Lee-Carter model to derive the iterative weighted least square method based on constraints to predict the mortality rate of the Chinese population. Wang, Zhao, and Chen [26] used multi-population joint modeling to predict the mortality of China’s high-age population. For the prediction of the elderly population with missing data, the most commonly used adjustment models in China are the Gomportz model, Weibull model, Logistic model, and Kannisto model. Zeng [27] by comparing various models to fit the elderly population in the four census data, it was found that Kannisto had the best effect. On this basis, some scholars [28,29] later used Kannisto model to predict the mortality of the elderly population by sex in China. There are historical data on China’s population mortality, with large fluctuations and poor data quality. There is a deviation between the observed mortality of a specific mantissa age and the actual mortality [30]. In view of this, this paper uses the Lee-Carter model to fit China’s mortality by age from 1997 to 2019 and uses Kannisto model to extend the age to 100 years, a more accurate mortality rate is obtained by fitting the data and predicting the parameters to obtain the life expectancy.

We investigated the possibility that the Lee-Carter model could be applied to missing data in China and proposed a single-factor model to explain all mortality dynamics. The current approach was not surpassed by more complex factor models, so we used the single-factor Lee-Carter model as the baseline model [31,32].

Combining its current problems with Chinese population data: In the Chinese census, which combines the population census, the 1% population sample survey and the annual 1% population change sample survey, death data are lacking compared to other aspects of data. Moreover, it is not highly standardized and faces the lack of time series data, and the lack of data for single age group population is becoming more and more prominent, and the lack of data is more obvious in the data for infant and elderly population. Thus, when using the Lee-Carter model, special attention should be paid to the treatment of missing data [33].

The improvements in this paper are as follows:The first is that the parameter estimation cannot be performed using matrix singular value decomposition because the individual age-specific mortality rates are missing or zero and cannot be logarithmic. In order to improve the prediction accuracy, this paper uses spline fitting to supplement the outliers and missing values and then uses maximum likelihood estimation method to estimate the parameter. The quality of Chinese senior population data is poor, and this paper uses the Kannisto method to extend the senior population data to improve the prediction accuracy.

The paper is structured as follows. Material and methods are described in Section 2. In Section 2, the first part gives the benchmark models of Lee-Carter and the indicators of mortality used in this analysis. The second and third part gives the estimation method of parameters. Section 3 gives estimated results of parameters and predicted mortality. Section 4 gives the impact of mortality improvements, making separate projections of life expectancy for both sexes and further analyzing the risk of longevity associated with increased longevity.

## 2. Materials and Methods

In this paper, the prediction of mortality is mainly divided into the following steps:

Step 1:

- Model classical algorithm Establish a logarithmic center mortality model and estimate the model parameters through past data. The basic formula of the model is:
  (1) $$ln\left(m_{x,t}\right)=\alpha_{x}+\beta_{x}\kappa_{t}+\varepsilon_{x,t}.$$
  We let $\kappa_{t}$ be the central rate of death at age *x* and in year *t*. where $\alpha_{x}$ and $\beta_{x}$ are age-specific parameters, *x* is a time-varying index, and $\varepsilon_{x,t}$ is a random item with zero means and finite variances, the variances is σ.
  Since there are numerous groups that meet the requirements of the equation, Lee and Carter have standardized the parameters to estimate the above parameters, we let $\sum_{x}^{}\beta_{x}=1$, $\sum_{t}^{}\kappa_{t}=0$, $\hat{\alpha }=\frac{1}{T}\sum_{t}^{}ln\left(m_{x,t}\right)$, $\kappa_{t}=\sum_{x}^{}\left[ln\left(m_{x,t}\right)-\alpha_{x}\right]$.
  where *t* is the total number of calendar years included in the mortality data to be estimated. $\alpha_{x}$ is the mean of logarithmic center mortality for age. $\kappa_{t}$ indicates the intensity of mortality at year. Derived from both sides of Equation (1).
  We obtain $\hat{\beta_{x}}=\frac{\partial ln\left(m_{x,t}\right)}{\partial t}\cdot \frac{\partial t}{\partial \kappa_{t}}$. That is, $\beta_{x}$ represents the sensitivity of logarithmic mortality change when the mortality intensity changes.
- Maximum likelihood estimation is used to estimate the parameters in Lee-Carter model. The method assumes that the number of deaths $d_{x,t}$ follows the Poisson distribution with parameter λx,t, that is:
  (2) $$D_{x,t}∼Possion\left(m_{x,t},E_{x,t}\right),m_{x,}=exp\left(\alpha_{x}+\beta_{x}\kappa_{t}\right).$$
  In Equation (3), $D_{x,t}$ is the annual mortality of the population aged *x* in year *t*, and $E_{x,t}$ is the death risk exposure of the population in the year of death. In practical application, it is often replaced by the number of people in this year $N_{x,t}$. By maximizing the following likelihood function, we can obtain an estimate of the sum.
  (3) $$L\left(\alpha ,\beta ,\kappa \right)=\sum_{x,t}\left(D_{x,t}\left(\alpha_{x}+\beta_{x}\kappa_{t}\right)-N_{x,t}\exp\left(\alpha_{x}+\beta_{x}\kappa_{t}\right)\right)+C.$$
  where *C* is a constant, the estimated values of parameters $alpha_{x}$, $beta_{x}$ and $kappa_{t}$ are given by Newton iterative method.
  (4) $${\hat{\theta }}^{\left(v+1\right)}=\hat{\theta }-\frac{\partial L^{\left(v\right)}/\partial \theta }{\partial^{2}L^{\left(v\right)}/\partial \theta^{2}}.$$
  where $\theta^{\left(v\right)}$ is the parameter of the *v*-th iteration, and $L^{\left(v\right)}=L^{\left(v\right)}\left(\theta^{\left(v\right)}\right)$ is the corresponding likelihood function. Therefore, the iteration formula of the sum of parameters is obtained.
  (5) $${\hat{\beta }}_{x}^{\left(v+1\right)}={\hat{\beta }}_{x}^{\left(v\right)}+\frac{\sum_{t}w_{x,t}\left(D_{x,t}-{\hat{D}}_{x,t}^{\left(v\right)}{\hat{\kappa }}_{t}^{\left(v\right)}\right)}{\sum_{t}w_{x,t}{\hat{D}}_{\left(x,t\right)}^{\left(v\right)}{\left({\hat{K}}_{t}^{\left(v\right)}\right)}^{2}},$$
  (6) $${\hat{\kappa }}_{x}^{\left(v+1\right)}={\hat{\kappa }}_{x}^{\left(v\right)}+\frac{\sum_{x}w_{x,t}\left(D_{x,t}-{\hat{D}}_{x,t}^{\left(v\right)}{\hat{\beta }}_{x}^{\left(v\right)}\right)}{\sum_{x}w_{x,t}{\hat{D}}_{\left(x,t\right)}^{\left(v\right)}{\left({\hat{\beta }}_{x}^{\left(v\right)}\right)}^{2}},$$
  (7) $${\hat{\alpha }}_{x}^{\left(v+1\right)}={\hat{\alpha }}_{x}^{\left(v\right)}+\frac{\sum_{x}w_{x,t}\left(D_{xt}-{\hat{D}}_{x,t}^{\left(v\right)}\right)}{\sum_{t}w_{x,t}{\hat{D}}_{\left(x,t\right)}^{\left(v\right)}}.$$
  where ${\hat{D}}_{x,t}^{\left(v\right)}=N_{x,t}\exp\left({\hat{\alpha }}_{x}^{\left(v\right)}+{\hat{\beta }}_{x}^{\left(v\right)}{\hat{\kappa }}_{t}^{\left(v\right)}\right)$ is the estimated value of the *v*-th death toll under the assumption of Possion distribution, where $w_{x,t}$ is the weight, $w_{x,t}=\left\{\begin{array}{cc} 1, & \;\mathrm{Data}\;\mathrm{missing},\;\\ 0, & \;\mathrm{Data}\;\mathrm{available}.\; \end{array}\right.$.
  After the above iterative process, $D\left(D_{x,t},{\hat{D}}_{xtt}\right)$ is small enough, Likelihood formula approaches maximum,
  where $D\left(D_{xt},{\hat{D}}_{x,t}\right)=\sum_{x,t}{\mathrm{dev}}_{p}\left(x,t\right)=\sum_{x,t}w_{xt}\left(D_{x,t}\log\frac{D_{x,t}}{{\hat{D}}_{x,t}}-\left(D_{x,t}-{\hat{D}}_{xt}\right)\right)$.

Step 2: predict $\kappa_{t}$.

- In the second stage, $\kappa_{t}$ is modeled and fitted according to the time series method. The model is very simple [34,35]. It only needs endogenous variables without the help of other exogenous variables. In most studies, ARIMA (p, d, q) process is used to model $\kappa_{t}$ series. ARIMA’s prediction model can be expressed as:
  (8) $$\hat{\kappa_{t}}=\mu *\phi_{1}+\kappa_{t-1}+\ldots +\phi_{p}*\kappa_{t-p}+\theta_{1}*e_{t-1}+\ldots +\theta_{q}*e_{t-q}.$$
  where ϕ represents the coefficient of AR, θ represents the coefficient of Ma. In this way, the time series method can be used to simulate the time effect $\hat{\kappa_{t}}$, and the value of the predicted year $\hat{\kappa_{t}}$ can be obtained by trend extrapolation. On this basis, the future mortality $m_{x},t$, is predicted, that is
  (9) $$ln\left(m_{x,t}\right)={\hat{\alpha }}_{x}+{\hat{\beta }}_{x}{\hat{\kappa }}_{t}.$$

## 3. Results

### 3.1. Data Description

#### 3.1.1. Data Source and Processing

The data set used in this paper is extracted from the China demographic and Employment Statistical Yearbook and the China demographic statistical yearbook from 1998 to 2020, including the death toll and random inspection data from 1997 to 2019. In the data set used in this paper, 2000 and 2010 are census data, 2005 and 2015 are 1% sampling data, and other years are population change sampling. According to the characteristics of the selected data and the need of empirical analysis, the specific data processing and assumptions are as follows:

1. In order to keep the number of deaths and the overall population in each year roughly in order of magnitude, divide the census data by 1000, and divide the 1% spot check data by 10. According to the mortality data of different ages, the death toll of each age is adjusted based on 1 million;
2. It is assumed that both 1% population sampling and variable sampling methods have good random sampling characteristics;
3. The data for most years are from 0 to 89 years old, and the data for 2000, 2005, 2010, and 2015 are from 0 to 99 years old. This paper uses kannisto method to fit the missing data of 90–100 years old in the data;
4. When the death toll data is zero or missing, the cubic spline interpolation method for the year is used to fill in.

#### 3.1.2. Descriptive

**Figure 1:**
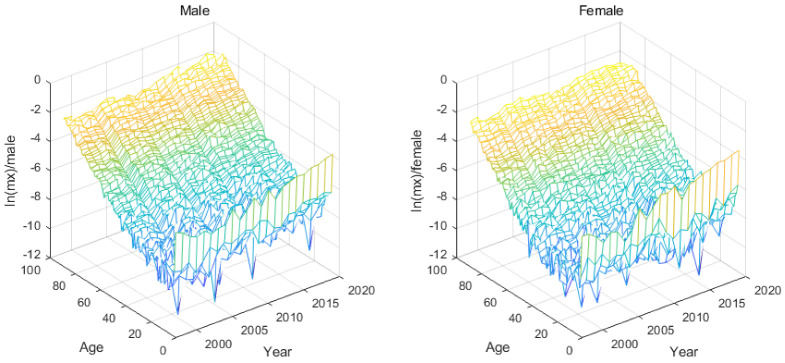
All age mortality of both sexes.

Figure 1 depicts the full age mortality rate for both sexes in 1997–2019 and the missing data is interpolated by the above method. From Figure 1, we can see the changes in mortality in recent years. The mortality of young children and the elderly fluctuates greatly, which interferes with the establishment of the model.

### 3.2. Parameter Estimation

**Figure 2:**
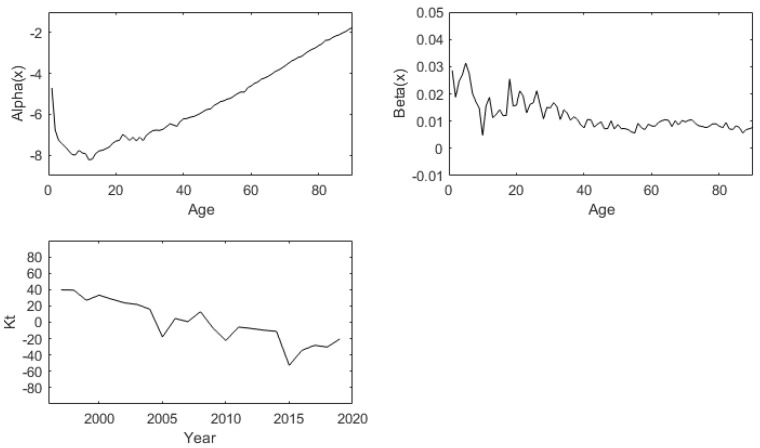
Fitted components of basic LC method for males of China (1996:2019).

**Figure 3:**
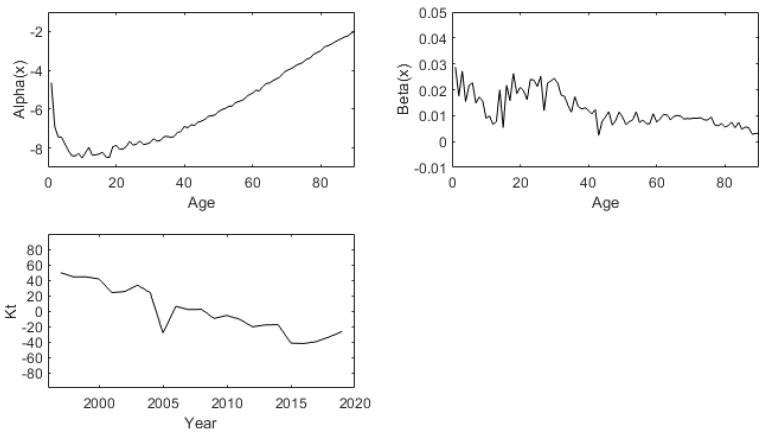
Fitted components of basic LC method for females of China (1996:2019).

**Figure 4:**
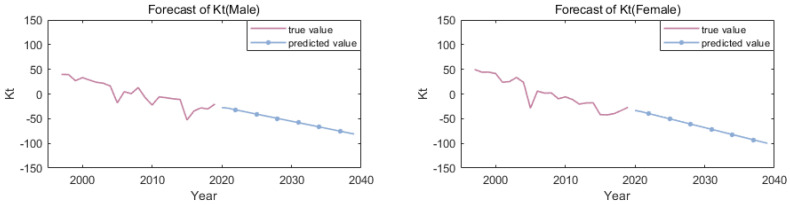
Observed (1997:2019) and 20 years ahead forecast (2020:2039) of $\kappa_{t}$ for males and females.

- Parameter of male:
  The weighted least square method (WLS) is used to estimate the parameter age effect factors α, β and time factor κ of Lee-Carter model, as shown in Figure 2 below:

- Parameter of female:
  The weighted least square method (WLS) is used to estimate the parameter age effect factors α, β and time factor κ of Lee-Carter model, as shown in Figure 3 below:

- Forecast value of $\kappa_{t}$.
  $\kappa_{t}$ is a time series model, the unit root AR(1) model is usually employed in the studying of longevity risk, which is a random walk process with drift term. But in this paper, by fitting the ARIMA model, using AIC criteria and t-test statistics, the optimal ARIMA model is selected. Both men and women are ARIMA (1, 2, 1).
  As discussed before, the parameter $\kappa_{t}$ is used for the forecast; the blue lines of $\kappa_{t}$ in Figure 4. Table 1 are the predicted results of $\kappa_{t}$ value.

### 3.3. Age Factor Expansion

There is a serious lack of data on the elderly population in China. Even the census data are underreported and concealed. The lack of data leads to large fluctuations in the curvature of the tail. In China’s Demographic Yearbook and China’s demographic and Employment Statistical Yearbook, there is a lack of population data over 90 years old. Therefore, this paper uses kannisto model to correct the data on the elderly population. By extending the age factor of the model, the limitation of the highest age mortality rate in China’s population data can be lifted, making the results more realistic.

The form of kannisto model is as follows:

(10) $$u_{x}=\frac{\alpha e^{\beta x}}{1+\alpha e^{\beta x}},$$

where $u_{x}$ is the death power, α and β are parameters to be estimated. In this paper, the mortality data of 60–89 years old are used for fitting, and the parameters α and β to be estimated are calculated to obtain the death power. Through the relationship between the death power and the probability of death and the central mortality, we can obtain the expanded mortality rate.

(11) $$u_{x}=-\ln\left(1-q_{x}\right),$$

(12) $$q_{x}=\frac{m_{x}}{1+0.5*m_{x}},$$

where $q_{x}$ is the probability of death and the $m_{x}$ is the central morality.

**Figure 5:**
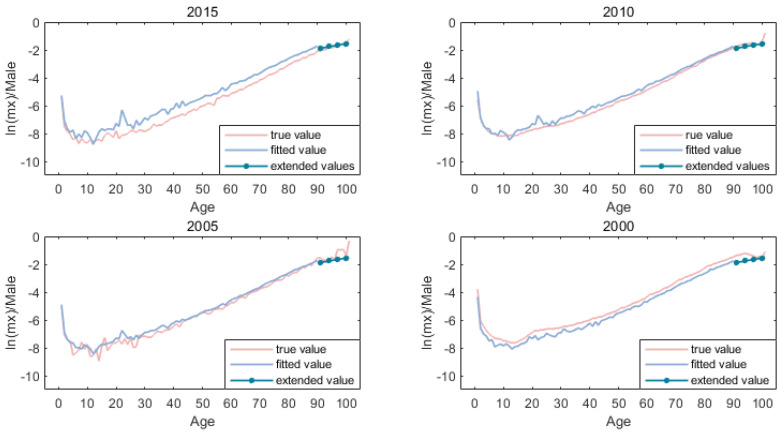
Mortality extension of male.

**Figure 6:**
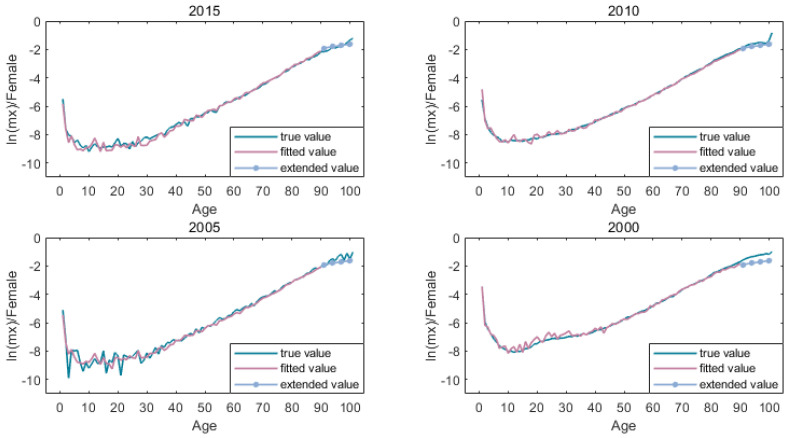
Mortality extension of female.

Figure 5 and Figure 6 shows the mortality rate of the high-age population after the expansion of the kannisto method.

### 3.4. Mortality Prediction

**Figure 7:**
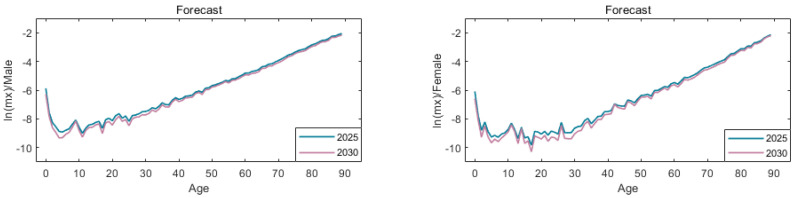
Comparison chart of predicted mortality.

According to the above methods, the mortality rates by age and sex in the future are shown in Figure 7 and Table 2. (Due to space limitations, this paper shows the mortality data by age and sex in 2025, and 2030, and only some age data are listed).

By comparing the mortality rates in 2019, 2025 and 2030, it can be seen that the mortality rate is continuously improving and the life expectancy is continuously extending. The difference between this and the future actual mortality rate will lead to the occurrence of longevity risk. At the same time, by comparing the changes of male and female mortality, the female mortality fluctuates greatly. In general, the risk of longevity in China is gradually increasing.

### 3.5. Model Evaluation

#### 3.5.1. Residual Plot Test

**Figure 8:**
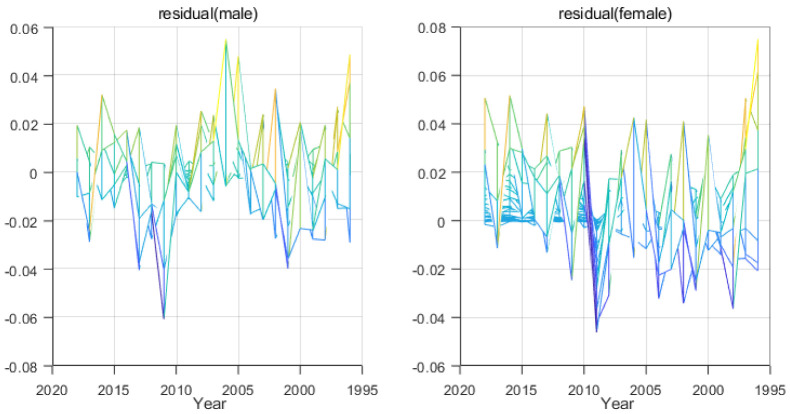
Residual Graph.

By analyzing the residual fit plots, we can visually analyze the model fitting effect and determine whether the model misses important influence information. Ideally, the residuals should be 0. Considering the realistic factors, they should be randomly distributed around 0. The following Figure 8 shows the distribution of residuals.

The residual distribution plot shows that the model fits better, and the distribution of residuals is more concentrated in men than in women, indicating that the fit is better than in women.

#### 3.5.2. MAPE Value Test

In the previous section, we obtained the prediction results for mortality, and to evaluate the model, we used the mean absolute percentage error(MAPE) to evaluate the model fit results.

$$\mathrm{MAPE}\left(\hat{y}\right)=\frac{1}{n}\sum_{i=1}^{n}\frac{\left|y_{i}-{\hat{y}}_{i}\right|}{y_{i}},$$

where $y_{i}$ is the true value, ${\hat{y}}_{i}$ is the predicted value, and *n* is the number of periods.

(13) $$\mathrm{MAPE}\left({\hat{m}}_{x,t}\right)=\frac{1}{10\times 100}\sum_{x=1}^{100}\sum_{t=1}^{10}\frac{\left|m_{x,t}-{\hat{m}}_{x,t}\right|}{m_{x,t}}.$$

The calculation results are shown in the following table.

Combined with the data in the Table 3, the deviation of the period forecast results is small and acceptable.

### 3.6. Conclusion

#### Impact on Life Expectancy

The average life expectancy of a cohort is calculated from the level of mortality in each year, using the level of mortality for each age of the population in the same year instead of the level of mortality for the same generation at different ages. Before using the mortality rate to find the average life expectancy, the mortality rate $m_{x,t}$ is converted into the probability of death $q_{x,t}$, and then the probability of death is used to calculate the average life expectancy.

(14) $$q_{x,t}=\frac{2m_{x,t}}{1+2m_{xt}},$$

Then life expectancy:

$$e_{x}\left(t\right)=\sum_{k=1}^{T-x}p_{x,\kappa }\left(t\right)+0.5,$$

which ${}_{\kappa }p_{x,\kappa }\left(t\right)=\prod_{i=0}^{x-1}\left[1-q_{x+i}\left(t\right)\right].$

The part of life expectancy projection results are shown in the following Table 4.

The short-term life expectancy prediction results given above using the Lee-Carter model, which has robust prediction results in the short term, but may not be robust in the long term with its narrower intervals.By comparing with the average life expectancy of Chinese population published by WHO, the error is small and the fitting result is good. However, we found that the average male life expectancy projection is higher and the average female life expectancy projection is lower. Moreover, the larger difference of female projections may be due to the lower quality of female population mortality data compared to male population mortality data. Therefore, in the subsequent projections, attention should be paid to the correction of the female crude mortality population. By comparing with the average life expectancy of Chinese population published by WHO, the error is small and the fitting result is good.

## 4. Discussion

Population health is a hot issue in today’s society, and it is also an issue that China should always pay close attention to In the process of China’s aging population, given the actual situation of China’s population, we should actively face a series of problems brought by the aging population, and deal with the challenges it brings. We must carry out basic research on China’s population situation. Mortality is an important indicator to measure the overall health status of a country’s population, which can directly reflect the quality of life of the population and is also an important basis for judging the social-economic, scientific, and cultural levels. The prediction of mortality can monitor the expected problems of China’s population, which is of great significance for responding to and implementing the strategy of actively dealing with aging.

Based on the data of the 1997–2019 national census, the 1% population sampling survey, and the annual 1‰ population change sampling survey, this study uses the Lee-Carter mortality prediction model to predict the mortality of both sexes in China. Through the study, the following conclusions can be drawn.

With the continuous development of society and the continuous improvement of the level of science and technology and medical treatment, on the whole, the death rate in all regions of China has improved significantly from 1997 to 2019, and at the same time, the life expectancy is also increasing. In terms of age, the mortality rate of each age has decreased, but the improvement of each age is very different. Among them, the mortality rate of the 0-year-old group and the elderly population has decreased the fastest. Changes in mortality are closely related to age. This paper does not group the age groups, and directly uses the data of each age from 0 to 89 years old. This method can obtain the information of the original data to a greater extent, but it also makes the mortality data more volatile and not smooth enough to a certain extent. Therefore, this paper fills and fits the missing data with spline interpolation, which can make the original data smoother. Second, this paper uses Kannisto to extend the data of the senior population, which makes the prediction of life expectancy more accurate. However, the prediction process in this paper also has some degree of drawback: the period of having higher quality population data in China is short, and using ARIMA model for Kt value prediction increases the prediction bias to some extent. More scenarios should be considered when predicting K values. Finally, from 2020 to 2030, the population mortality rate, whether male or female, will decline significantly, and the decline of age-specific mortality rate will affect people’s life expectancy, which will increase the pressure of social aging to a certain extent, bring public financial burden to the government, but also bring risks to insurance companies and individuals. With the improvement in mortality and the increase in life expectancy, the government needs to increase pensions. Therefore, the government should improve the operation of pension insurance funds and balance revenue and expenditure. For insurance companies, when formulating the rates of different insurance products, based on referring to the life table, they should also take full account of the risks brought by the improvement of mortality and reasonably design insurance policies.

In conclusion, through the prediction of mortality, the accurate prediction of mortality and population structure will help the decision-making level to formulate population policies, economic policies, and pension policies in line with the future population characteristics, and weaken the negative impact of population aging.

## 5. Conclusions

Population mortality is an important step in quantifying the risk of longevity. There is a lack of data on population mortality in China, especially for the elderly population. In this paper, we first use spline fitting to supplement missing data, and then use Lee-Carter dynamic models to predict population mortality in China, including age extrapolation and trend extrapolation. This paper improves the accuracy of population mortality prediction by filling the gap in the original data, and also predicts the life expectancy of the Chinese population, and the results can provide a theoretical basis for policies to address the risk of longevity.

## Figures and Tables

**Table 1:** This is the prediction of $\kappa_{t}$.

$\kappa_{t}$ (Year)	Male	Female
1997	39.71694	49.62551
1998	39.37878	44.25081
1999	26.90062	44.27759
2000	33.22122	41.42727
2001	28.28746	23.72562
2002	23.76912	25.18467
2003	21.81028	33.53104
2004	16.12240	23.64792
2005	−18.00578	−28.60565
2006	4.64959	5.93254
2007	0.74380	1.80809
2008	13.01404	2.12542
2009	−7.32940	−9.68799
2010	−22.27087	−5.76496
2011	−5.84174	−10.94736
2012	−7.58277	−20.49683
2013	−9.67512	−18.16568
2014	−11.03544	−17.79088
2015	−52.66777	−41.73031
2016	−34.43803	−42.25345
2017	−28.12194	−39.70408
2018	−30.28050	−33.61352
2019	−20.36489	−26.77579
2020	−27.36053	−33.40219
2021	−28.92028	−36.01515
2022	−32.22732	−39.82445
2023	−34.97272	−43.27714
2024	−37.89865	−46.83612
2025	−40.76655	−50.36343
2026	−43.65310	−53.90017
2027	−46.53366	−57.43410
2028	−49.41614	−60.96887
2029	−52.29801	−64.50340
2030	−55.18007	−68.03799
2031	−58.06208	−71.57256
2032	−60.94410	−75.10714
2033	−63.82611	−78.64172
2034	−66.70813	−82.17630
2035	−69.59014	−85.71088
2036	−72.47216	−89.24546
2037	−75.35418	−92.78003
2038	−78.23619	−96.31461
2039	−81.11821	−99.84919

**Table 2:** Prediction results of mortality by sex and age.

Prediction	2025 Male	2030 Male	2025 Female	2030 Female
0	0.002815632	0.001864309	0.002279284	0.001372894
1	0.000529425	0.000404437	0.000421002	0.000309032
2	0.000259631	0.000182212	0.000151838	0.000094000
3	0.000193766	0.000131181	0.000270073	0.000205789
4	0.000140375	0.000089400	0.000137687	0.000093800
5	0.000090700	0.000094500	0.000063300	0.000134991
6	0.000152940	0.000114613	0.000106773	0.000082200
7	0.000168450	0.000131642	0.000093800	0.000069200
8	0.000232580	0.000187947	0.000115986	0.000088100
9	0.000309759	0.000289486	0.000126814	0.000108278
10	0.000189158	0.000151044	0.000160350	0.000134703
11	0.000175596	0.000149353	0.000248531	0.000220986
12	0.000221387	0.000185156	0.000156758	0.000136913
13	0.000230460	0.000187790	0.000085925	0.000060314
14	0.000262990	0.000221228	0.000188295	0.000171488
15	0.000282440	0.000237320	0.000089595	0.000060976
16	0.000175137	0.000121344	0.000096146	0.000072854
17	0.000319452	0.000255342	0.000054329	0.000034126
18	0.000349785	0.000278339	0.000140665	0.000101496
19	0.000295161	0.000217803	0.000134585	0.000092890
20	0.000420180	0.000318750	0.000116659	0.000082486
...	...	...	...	...

**Table 3:** MAPE indicators.

	Male	Female
MAPE	0.49198	0.43572

**Table 4:** Life expectancy on average.

	2018	2019	...	2023	2024	2025	...
male	74.878	75.227	...	76.729	77.024	77.308	...
true (male)	73.64	74.7	...	—	—	—	...
female	78.717	79.694	...	82.23	82.17	82.24	...
true (female)	79.43	80.5	...	—	—	—	...

## Data Availability

The data set used in this paper is extracted from the China population and Employment Statistical Yearbook and the China population statistical yearbook from 1998 to 2020, including the death toll and random inspection data from 1997 to 2019.
